# Vitamin D supplementation compared to placebo in people with First Episode psychosis - Neuroprotection Design (DFEND): a protocol for a randomised, double-blind, placebo-controlled, parallel-group trial

**DOI:** 10.1186/s13063-019-3758-9

**Published:** 2020-01-06

**Authors:** Fiona Gaughran, Dominic Stringer, Michael Berk, Shubulade Smith, David Taylor, Eromona Whiskey, Sabine Landau, Robin Murray, Philip McGuire, Poonam Gardner-Sood, Gabriella Wojewodka, Simone Ciufolini, Harriet Jordan, Jessie Clarke, Lauren Allen, Amir Krivoy, Brendon Stubbs, Philippa Lowe, Maurice Arbuthnott, Shanaya Rathod, Andrew Boardman, Mudasir Firdosi, John J. McGrath

**Affiliations:** 1 Department of Psychosis Studies, King’s College London, Institute of Psychiatry, Psychology and Neuroscience, 16 De Crespigny Park, London, SE5 8AF UK; 2 South London and Maudsley NHS Foundation Trust, Denmark Hill, London, SE5 8AZ UK; 3 Department of Biostatistics and Health Informatics, King’s College London, Institute of Psychiatry, Psychology and Neuroscience, 16 De Crespigny Park, London, SE5 8AF UK; 4 Deakin University and Barwon Health, Ryrie Street, Geelong, Victoria 3220 Australia; 5 Carer Expert and Chair of Trustees, Rethink Mental Illness, 89 Albert Embankment, London, SE1 7TP UK; 6 London, UK; 7 Clinical Trials Facility, Research Department, Tom Rudd Unit, Moorgreen Hospital, Southampton, SO3 03J UK; 8 Cheshire & Wirral Partnership NHS Trust, Churton House, Countess of Chester Health Park, Chester, CH2 1BQ UK; 9 South West London and St George’s Mental Health NHS Trust, Queen Mary’s Hospital, Roehampton Lane, London, SW15 5PN UK; 10 Queensland Centre for Mental Health Research, The Park Centre for Mental Health, Wacol, QLD 4076 Australia; 11 Queensland Brain Institute, University of Queensland, Brisbane, QLD 4072 Australia; 12 National Centre for Register-Based Research, Aarhus University, 8000 Aarhus C, Denmark

**Keywords:** Psychosis, First episode, Vitamin D, 25OHD, Randomised controlled trial, Positive and Negative Syndrome Scale, Mental health

## Abstract

**Background:**

People experiencing their first episode of psychosis are often deficient in vitamin D. Observational studies have reported an association between low vitamin D concentrations and poorer subsequent health outcomes in psychosis. A vitamin D deficiency in neonates and children has been linked to a later increased risk of schizophrenia and psychotic-like experiences. This trial aims to examine the effect of high-dose vitamin D supplementation on outcomes in early psychosis. We hypothesise that vitamin D supplementation will be associated with better mental health outcomes.

**Methods/design:**

The DFEND study is a multicentre double-blind placebo-controlled parallel-group trial of vitamin D supplementation in people with early psychosis. Patients with an ICD-10 diagnosis of functional psychosis will be randomised in a 1:1 ratio to receive either 120,000 IU/month of vitamin D (cholecalciferol) or a matched placebo for 6 months. The primary outcome is the total Positive and Negative Syndrome Scale (PANSS) score at the 6-month follow-up for all patients. Secondary outcomes include assessment of mood (Calgary Depression Scale), general function (Global Assessment of Functioning), cardiovascular risk (body mass index, waist circumference, C-reactive protein, cholesterol and HbA1c) and vitamin D levels at the 6-month follow-up. Additionally, 3- and 6-month total PANSS scores will be analysed for those with inadequate vitamin D levels at the baseline.

**Discussion:**

The DFEND study is the first trial to examine whether vitamin D supplementation in early psychosis is associated with better mental health outcomes. The findings of this study may help to resolve the clinical equipoise regarding the benefits and cost-effectiveness of routine vitamin D supplementation in people with psychosis.

**Trial registration:**

ISRCTN, ISRCTN12424842. Registered on 25 February 2015.

## Background

Vitamin D is a fat-soluble secosteroid obtained largely through sun exposure and, to a lesser extent, food sources such as oily fish (e.g. mackerel) and egg yolk. The best-known role of vitamin D is in regulating bone health, being essential for the regulation of calcium in the body, but latterly, important additional functions have been identified. These include links between brain function and vitamin D [1, 2] with in vitro and animal experiments demonstrating a neuroprotective effect [3]. In rodents, developmental vitamin D deficiency alters a wide range of outcomes in the adult brain, including levels of neurotransmitters relevant to schizophrenia [4, 5], while vitamin D deficiency during adulthood has been associated with changes in behaviour and brain neurochemistry [6]. In animal models, vitamin D is neuroprotective, preventing neuronal damage from inflammation and oxidative stress [7] and inducing nerve growth factor [8]. Greater ischaemic brain damage due to a stroke is seen in rodents deficient in vitamin D [9]. The long-term adverse neurobehavioural effects of maternal immune activation in offspring can be attenuated by administration of 1-, 25-hydroxyvitamin D_3_ [10]. With respect to neuropsychiatric disorders, a randomised control trial of vitamin D supplementation in those with established Parkinson’s disease showed a slower progression compared with a placebo over a 12-month follow-up [11]. Based on these findings, it has been hypothesised that vitamin D deficiency during adulthood may worsen brain-related outcomes in those with prior neuropsychiatric disorders [12].

Schizophrenia is more common in people born in the winter and spring months [13] and at higher latitudes [14], both conditions in which exposure to the sun, and so vitamin D production, is limited. In addition, dark-skinned individuals living in cold (i.e., less sunny) countries have an increased risk of schizophrenia [15]. McGrath et al. hypothesised that low levels of vitamin D in early life may be linked to an increased risk of later development of schizophrenia [16]. Indeed, a large Danish study found a higher relative risk of developing schizophrenia in adult life in neonates with low vitamin D levels [17]. Similarly, low total vitamin D_3_ levels during childhood are associated with a higher risk of psychotic experiences during adolescence [18]. A recent meta-analysis found an overall prevalence of vitamin D deficiency in patients with schizophrenia of 65% and that people with vitamin D deficiency were 2.16 times more at risk of developing the condition [19]. Diet appears to have an effect also. A large population-based study of Swedish women (*n* = 33,623) reported an association between low dietary vitamin D intake and an increased risk of psychotic-like experiences [20]. The evidence, therefore, suggests that low levels of vitamin D disrupt early brain development and may also influence later brain function.

Vitamin D deficiency is commonly defined in the UK as levels of 25-hydroxyvitamin D (25OHD) less than 25 nmol/L, insufficiency as between 25 and 50 nmol/L and sufficiency as greater than 50 nmol/L [21]. Those with established psychosis in the UK are highly likely to be vitamin D deficient, with only 14% having sufficient vitamin D levels [22]. This is true even in the early stages of psychosis. Crews et al. examined the vitamin D status of 69 people after their first episode of psychosis (FEP) along with 69 controls of similar age, sex and ethnicity [23]. Over a third had a vitamin D deficiency, while an additional 25% had a vitamin D insufficiency. Overall, the people with FEP had a nearly threefold greater risk of vitamin D deficiency than comparators. Higher vitamin D levels at presentation were associated with lower total and negative symptoms of psychosis a year later [24]. The deficiency in vitamin D seems to be unrelated to other vitamins and minerals. A meta-analysis of nutritional deficiencies in FEP produced the strongest evidence for vitamin D deficiency and some evidence for low levels of vitamin C and folate in FEP, but no evidence for deficiencies in vitamins A and E, or in minerals [25].

People with severe mental illnesses, such as schizophrenia and schizoaffective disorder, experience poor physical health and high rates of premature death [26]. They have higher levels of cardiometabolic risk factors, including higher rates of diabetes, hypertension and central obesity [27–29]. Low vitamin D levels have been associated with cardiometabolic risk in both the general population [30, 31] and in people with established psychosis [22]. Large population-based placebo-controlled studies are currently underway that will examine the influence of vitamin D supplement on general health including cardiometabolic outcomes [32].

Despite research suggesting that vitamin D is linked to brain function and schizophrenia, little high-quality evidence is available on the impact of vitamin D supplementation on outcomes for people with psychosis. Whereas several studies have given vitamin D supplements to people with psychosis [33–35], these largely focused on physical health outcomes and 25OHD concentrations and had uncontrolled research designs. However, one recent randomised controlled trial in Israel explored whether vitamin D supplementation of 14,000 IU per week for 8 weeks improved outcomes in patients with chronic schizophrenia who were being treated with clozapine, but found no improvement in mental health, cognitive or metabolic outcomes [36]. To our knowledge, there have been no trials examining vitamin D supplementation in FEP. If vitamin D confers a positive neuroprotective effect and improves outcomes in psychosis, the first episode may be the ideal window of opportunity.

Clinical equipoise, therefore, persists as to whether routine vitamin D supplementation in early psychosis can improve mental or physical health outcomes. This paper describes the study protocol for the DFEND study, which is a randomised double-blind placebo-controlled parallel-group trial evaluating the impact of 6 months of vitamin D supplementation on Positive and Negative Syndrome Scale (PANSS) outcomes in people with early psychosis.

## Method/design

### Aims and objectives

The primary objective of the trial is to determine whether adding a monthly supplementation of 120,000 IU of vitamin D_3_ (cholecalciferol) to the standard treatment is more efficacious than a placebo in improving outcomes (PANSS) at the 6-month follow-up after an FEP. Secondary objectives are to examine the total PANSS score at 3 months, PANSS sub-scores at 3 and 6 months, along with a broader range of clinically relevant outcomes including Global Assessment of Function (GAF), the Calgary Depression Scale (CDS), cardiovascular risk markers and 25OHD concentrations. Here we report the DFEND study protocol according to SPIRIT guidelines (see Supplementary Material Additional file 1 for the SPIRIT Checklist).

### Setting

The study will take place in facilities run by English mental health trusts, which are part of the National Health Service (NHS) in the United Kingdom. Participants will be identified and recruited from clinical mental health services, including early intervention services, home treatment teams, general and forensic inpatient units, and outpatient community teams. The study is sponsored jointly by Kings College London (London, UK) and the South London and Maudsley NHS Foundation Trust (London, UK).

### Ethics

The trial will be conducted in compliance with the principles of the Declaration of Helsinki (1996), the principles of Good Clinical Practice laid out by the International Conference on Harmonisation (ICH-GCP) [37] and in accordance with all applicable regulatory requirements including but not limited to the Research Governance Framework and the Medicines for Human Use (Clinical Trial) Regulations 2004, as amended in 2006 and any subsequent amendments.

The protocol and related documents have been approved centrally by the National Research Ethics Committee, London Dulwich (reference 14/LO/1588), and has received clinical trial authorisation from the Medicines and Healthcare Products Regulatory Agency (MHRA) (reference 14523/0261/001–004). Recruitment will not begin at study sites until local approvals have been obtained. It has also been approved by the Health Research Authority (HRA, reference IRAS 147978) and registered online (ISRCTN12424842, 10.1186/ISRCTN12424842, registered on 25 February 2015, accessed 28 January 2019).

### Eligibility criteria

The study population is patients experiencing FEP, defined as presenting to health services with symptoms of psychosis within the last 3 years. A minimum level of symptoms or PANSS score is not required to take part, and patients can be enrolled during a period of stable remission. Patients must have the capacity to give written consent, which is determined by the delegated team members in conjunction with treating clinicians if needed.

#### Inclusion criteria

- Aged between 18 and 65 years old, including women of child-bearing age
- Having a diagnosis of functional psychosis defined according to ICD-10 criteria for psychosis (codes F20–29 and F30–33)
- Willing to refrain from taking multivitamin or non-study vitamin D supplements that exceed 400 IU per day throughout the study
- Willing to give a blood sample to assess vitamin D levels at baseline
- Able to and have given written informed consent

#### Exclusion criteria

- Known intolerance of vitamin D_2_ or D_3_ or known allergy to any of the trial medications
- Taking vitamin D supplements at a dose exceeding 400 IU per day
- Having taken cardiac glycosides, calcium channel blockers or oral, intramuscular, or intravenous corticosteroids, bendroflumethiazide, isoniazid or rifampicin in the past month
- Known active tuberculosis, sarcoidosis, hypo- or hyperparathyroidism, past or present nephrolithiasis (renal stones), suspected or diagnosed hepatic or renal dysfunction, any malignancy other than non-melanoma skin cancer not in remission for ≥3 years, or calcium disorders
- Baseline corrected serum calcium >2.6 mmol/L
- Known history of hypercalcaemia
- Pregnant or breast-feeding women, or women planning a pregnancy
- Lacking the capacity to provide written informed consent
- Insufficient English to complete the core assessments with the available assistance

### Outcomes

**Fig. 1:**
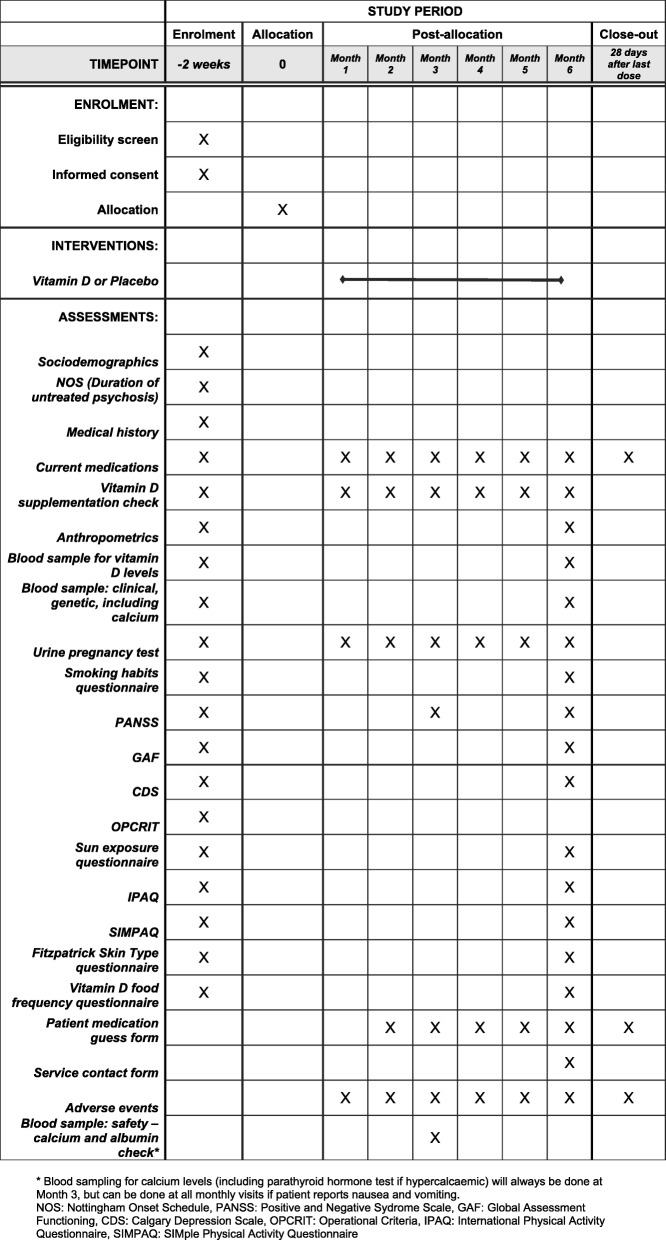
SPIRIT figure. Schedule of enrolment, interventions, and assessments in the DFEND Study. *Blood sampling for calcium levels (including a parathyroid hormone test if hypercalcaemic) will always be done at month 3 but can be done at all monthly visits if patient reports nausea or vomiting. NOS Nottingham Onset Schedule, PANSS Positive and Negative Syndrome Scale, GAF Global Assessment Functioning, CDS Calgary Depression Scale, OPCRIT Operational Criteria, IPAQ International Physical Activity Questionnaire, SIMPAQ Simple Physical Activity Questionnaire

All outcomes and time points are specified in Fig. 1.

#### Primary outcome

The primary outcome is symptom severity, as assessed via total PANSS scores at 6 months after randomisation [38]. PANSS assesses symptom severity across three domains: positive symptoms (seven items), negative symptoms (seven items) and general psychopathology (16 items) [38]. A total PANSS score is derived by summing scores across the three domains. Scores range from 30 to 210, with higher scores depicting greater symptom severity. In terms of reliability, α = 0.73, 0.83 and 0.79 for the positive, negative and general psychopathology scales, respectively [38]. All researchers will undergo two thorough stages of in-house PANSS training. Firstly, trainee researchers will watch and rate two PANSS training videos on two separate occasions, followed by feedback and discussion with the trial manager. Secondly, trainee researchers will shadow an experienced member of the team and observe real-world PANSS interviews, after which they will rate the interview to become familiar with the rating scale. Researchers will then be permitted to conduct the PANSS assessments independently. Researchers will also be required to attend a training session every six months to maintain their reliability throughout the trial.

#### Secondary outcomes

The following clinically relevant outcome measures will be compared between the trial arms:

- PANSS at 3 months
- PANSS sub-scores at 3 and 6 months (Positive Scale, Negative Scale and General Psychopathology Scale)
- GAF [39, 40] at 6 months: the assessment is used to rate the social, occupational and psychological functioning of adults. It is divided into two ratings, symptoms and disability, with a score for each. Reliability as measured by intra-class coefficients ranges from 0.81 to 0.94 [41–43]
- CDS [44] at 6 months, which rates symptoms of depression on a nine-point scale (α = 0.79) [45]
- Cardiovascular risk factors at 6 months: waist circumference, body mass index, and levels of C-reactive protein (CRP), haemoglobin A1c (HbA1c) and total cholesterol

We will examine all these outcomes for (a) all participants and (b) a subgroup of patients with suboptimal vitamin D concentrations at baseline. We will also measure the efficacy of vitamin D supplementation at 6 months as indicated by the 25OHD blood concentration.

Exploratory measurements include inflammatory and immune markers as well as genetic markers, processed in collaboration with the BioResource for Mental and Neurological Health based at the National Institure for Health Research (NIHR) Maudsley Biomedical Research Centre (London, UK). Participants will be asked to give written consent to allow their blood specimens to be stored for future research, which will be subject to new ethics approvals.

Outcomes will be assessed by objective face-to-face interviews, from information in medical notes or by self-report. All participants will complete outcome measures at baseline, 3 and 6 months (Fig. 1).

### Participant timeline

**Fig. 2:**
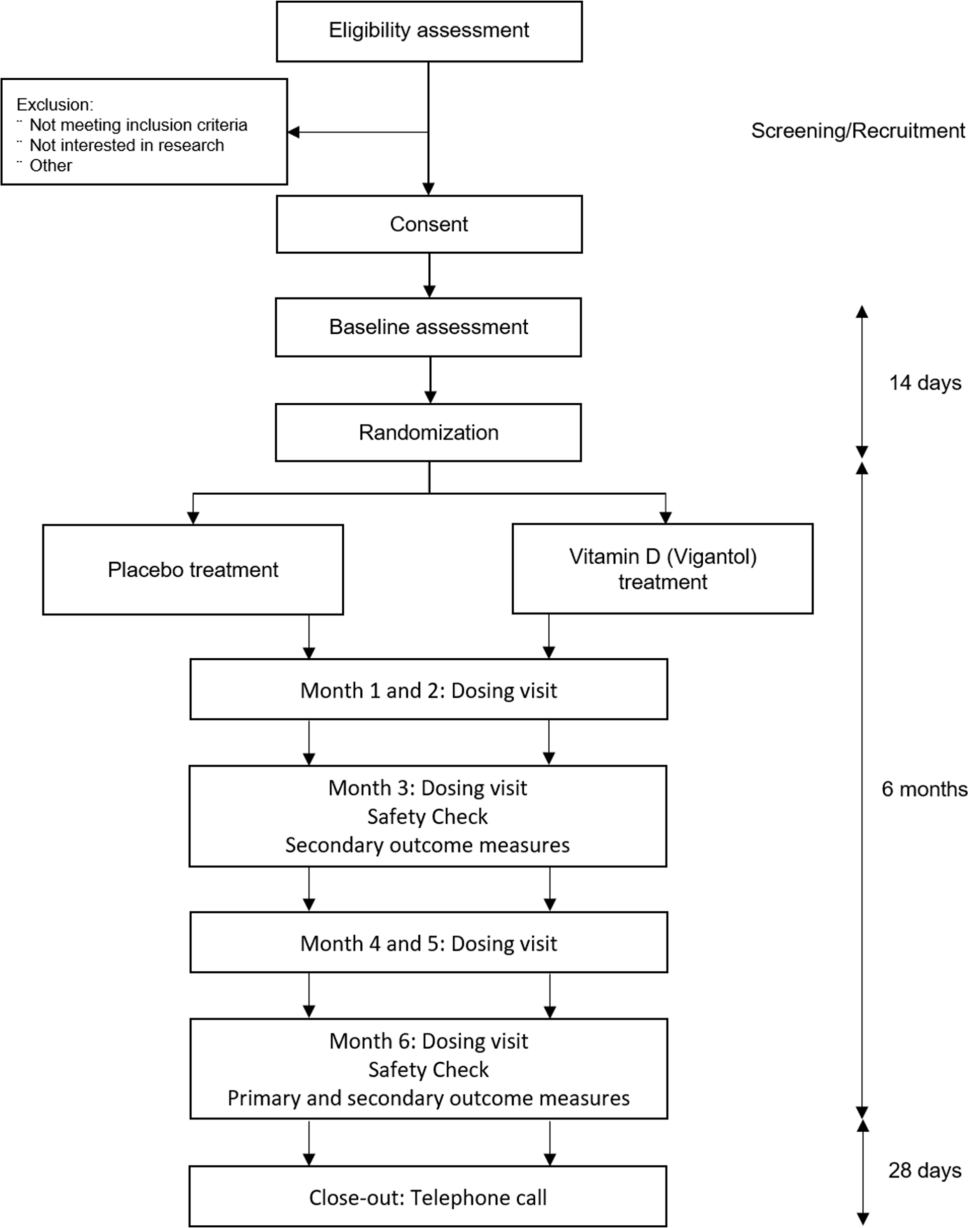
Participant flowchart.

Following consent and confirmation of eligibility, participants will be randomised to their treatment allocation within 14 days of the baseline assessment. They will attend 6 monthly visits to receive the dose of IMP or placebo. Final outcome measures will be assessed at the Month 6 visit. Each patient will receive a telephone call 28 days after their last dose of IMP/placebo as part of the close-out. The end of the trial will be defined as the last patient last close-out (Fig. 2).

### Sample size

Based on the number of new cases presenting to FEP services and annual counts of FEP patients recruited into similar studies, we predicted a recruitment and randomization rate of 120 patients per year for a total of 240. For the primary outcome (measured at the 6-month follow-up), we assumed a 20% attrition rate. Thus, the effective sample size is 192 (96 in each trial arm).

In the PUMP Study [46] (*n* = 190), the mean total PANSS score (and standard deviation) for patients with FEP was 58.1 (15.0). For the power analyses, we modelled two plausible scenarios using 80% and 90% power, respectively, with alpha = 0.05 for both scenarios. Based on alpha = 0.05, power = 80%, and samples with between 200 and 180 participants, we will confidently be able to detect group differences in mean total PANSS score of between 6 and 6.3. This equates to a standardised effect of size of approximately 0.4–0.42. Considering 90% power with the same power assumptions, we will be able to detect group differences in mean total PANSS score of between 6.9 and 7.3 (standardised effect size of 0.46–0.49).

As a secondary objective, we also wish to estimate the average treatment effect in those participants with a vitamin D deficiency at baseline. We assumed that 60% of the FEP population will have vitamin D concentrations below 50 nmol/L at baseline [23]. After 20% attrition at 6 months of the 60% who meet the vitamin D threshold, this will result in a projected sample size of 115. Based on alpha = 0.05, power = 80%, and samples with between 120 and 100 participants, we will confidently be able to detect differences in trial arms for mean total PANSS scores between 7.7 and 8.5 (effect size of 0.52–0.57). Considering 90% power with the same power assumptions, we will be able to detect group differences in mean total PANSS score between 8.95 and 9.8 (effect size of 0.60–0.65).

### Recruitment

To be eligible for entry into the trial, individuals who have experienced their first presentation to health services with psychosis within the last 3 years must satisfy all eligibility criteria. Researchers will visit wards and community services to speak with staff and will meet interested patients face to face or contact them by telephone. Once a patient has agreed to meet for a first appointment, a clinician (i.e. a doctor, research nurse or allied health professional) trained in ICH-GCP will be responsible for obtaining written informed consent. Eligibility will be confirmed by an ICH-GCP trained doctor for all trial participants prior to randomisation. Recruitment strategies will involve liaising with consultants throughout the trust to inform them about the study, visiting in-patient wards, attending physical health clinics in community settings and reaching out to care coordinators about their patients.

### Randomisation

An online randomisation service will be provided by King’s Clinical Trials Unit (King’s College London, London, UK). Randomisation will take place after collection of baseline measures. Randomisation will be stratified according to ethnicity (white or other) at a 1:1 ratio with randomly varying block sizes of 2–4. While there are no known predictors of PANSS scores in our target population, the concentration of vitamin D in blood varies by ethnicity [47], and we hypothesize that this concentration mediates any effect of vitamin supplements.

### Intervention

All eligible patients will be randomised to receive once per month either 120,000 IU of vitamin D_3_ (cholecalciferol) (equivalent to 4000 IU per day) or placebo for a maximum of 6 months in a 1:1 ratio. We selected monthly dosing to help with adherence because this clinical group may already have a high tablet burden and are routinely assessed at intervals.

Vitamin D will be given as the drug Vigantol®, which contains vitamin D_3_ (cholecalciferol) in oil. The placebo will be an organoleptically matched triglyceride oil (Miglyol® 812 oil). Both the active medication and the placebo will be packaged in identical sealed glass bottles (fill volume of 8 mL). They will be administered orally as a 6 mL dose given in a graduated oral syringe by a trained researcher. Participants will receive a dose of either active medication or placebo at each monthtly visit over 6 months for a total of six doses. 

Guy’s and St Thomas’ Pharmacy Manufacturing Unit (London, UK) is responsible for arranging the manufacture of the investigational medicinal product (IMP), randomised labelling, packaging and final release by a qualified person for clinical trial use.

No restrictions are placed on prior or concomitant interventions (medications or therapies, rescue or subsequent treatments), other than that we ask the participants to refrain from taking vitamin D supplements (including multivitamins that contain vitamin D) that exceed 400 IU/day for the duration of the trial. This will be checked at baseline and each monthly visit.

### Assignment of intervention

Each bottle of IMP, whether the active medication or placebo, has a unique identification number. Each participant is assigned a unique identification number, which will be used throughout the duration of their treatment. When a participant’s identification number is entered into the randomization system, the algorithm will randomly allocate them to a study arm and notify the unblinded members of staff. At each monthly visit, pharmacy staff will assign a specific bottle of IMP to the participant. Only pharmacy staff dispensing the IMP and the pharmacy monitor (a clinical research associate) will be unblinded. The blinded members of staff include all research team members at site level including the chief investigator, each study site’s principal investigator, researchers undertaking assessments at each time point and trial statisticians. Participants will remain in their allocated treatment arm for the duration of the study, even if they discontinue taking their IMP.

### Emergency unblinding

Emergency unblinding and medical information will be provided by ESMS Global Ltd (London, UK). Each randomised subject will be given a card with telephone numbers to use for unblinding and emergency contact details. Subjects will be requested to carry this card with them at all times whilst participating in the trial. Only healthcare professionals who have direct responsibility for the care of trial participants, clinical trial investigators, pharmacists and the staff of King’s Health Partners Clinical Trials Office are authorised to request unblinding.

### Data collection and management

The trial data will be held in a customised electronic database (Infermed MACRO, Elsevier Ltd, version 4, London, UK), created and maintained by King’s Clinical Trials Unit. All source data will be collected directly on paper worksheets and then entered into an online case report form. The data entry screens will resemble the paper forms as closely as possible to minimise transcription errors. MACRO allows for data entry checks thereby enhancing data accuracy. Data can be extracted from the database for checks and analysis. All personal information collected on DFEND trial participants and on potential participants will be treated as confidential and will be handled according to good clinical practice guidelines, associated standard operating procedures, the Data Protection Act 1998 and the European General Data Protection Regulations [48]. All electronic files containing personal information will be held only on password-protected computers. The computers themselves will be kept securely. All other trial information, such as the trial master file, source notes and data code definitions, will be stored in a lockable fireproof facility that can be accessed only by delegated DFEND trial staff.

### Statistical analysis

Full details of the statistical analyses will be specified in a statistical analysis plan written in collaboration with the trial statisticians.

Descriptive analyses, recruitment rate, consent rate, loss to follow-up, departures from randomised treatment and the prevalence of serious adverse events post-randomisation will be reported and summarised by treatment arm over the course of the study. All causes of withdrawal from the randomised treatment will be reported.

Analyses will be carried out by the trial statistician, who will remain blinded until all analyses are completed. The analysis of vitamin D concentrations in blood samples taken at 6 months, which is a secondary outcome, will be carried out after all other analyses are completed, as there is a high expectation that the mean concentration will be higher in the intervention group.

An intention-to-treat analysis of the effect of vitamin D supplementation on the primary PANSS outcome will be conducted using linear mixed modelling. All the available follow-up data from the randomised participants will be modelled. The analysis model will include treatment arm (vitamin D or placebo), time (3 or 6 months), baseline PANSS and randomisation stratifier (ethnicity) as explanatory variables. The model will also contain a subject-varying random intercept to account for any correlation between the repeated measures. Treatment effects on the other secondary outcomes at 6 months will be assessed using similar modelling techniques, employing generalisations to non-normal data where necessary.

To address the secondary research objectives, we will also estimate treatment effects for the subpopulation who were vitamin D insufficient at baseline in terms of primary and secondary outcomes, except for 25OHD blood concentrations, by including relevant interaction terms between baseline vitamin D insufficiency status and treatment group in the statistical models.

We expect that some data will be missing in the post-treatment outcome variables. The linear mixed modelling analyses are based on maximum likelihood and will provide valid inferences under a missing-at-random missingness mechanism. We will explore predictors of missingness. If deemed suitable for adjustment, we will include these as explanatory variables in the analyses. If post-randomisation variables are identified, a multiple imputation model will be considered instead [49].

No interim analysis will be carried out. There are no formal prespecified stopping rules.

### Data monitoring

In accordance with ICH-GCP guidelines, the DFEND investigators will support regular trial-related monitoring and quality assurance audits by providing direct access to source data documents. King’s Health Partners Clinical Trials Office will monitor the study at all sites. All research staff will be advised to ensure that a thorough audit trail is maintained throughout the trial.

### Oversight committees

Regular meetings will take place with the data monitoring committee, comprising an independent chair, an independent statistician and an independent psychiatrist. This committee will safeguard the interests of DFEND trial participants, periodically review and evaluate the safety of the interventions during the trial, monitor the overall conduct of the trial and make recommendations to the trial steering committee (TSC) concerning the continuation, modification or termination of the trial. The data monitoring committee will also advise the chief investigator and trial management group to protect the validity and credibility of the DFEND trial.

The TSC has four independent members: two independent psychiatrists (one acting as chair) and two patient-and-public representatives. In addition, the DFEND chief investigator and a co-investigator sit on it. The TSC provides overall supervision of the trial, reporting on behalf of the sponsor and funder. The TSC ensures that the trial runs according to rigorous standards by monitoring the safety of participants, the progress of the trial, protocol adherence, patient safety and relevant information that could impact the trial aims. The TSC also advises the investigators on all aspects of the trial.

### Safety monitoring

An adverse event is defined as any untoward medical occurrence in a subject to whom a medicinal product has been administered, including occurrences that are not necessarily caused by or related to that product. Any adverse event that occurs between the time of consent to the study through to 28 days following the last dosing visit will be recorded. The principal investigators and study doctors will assess whether the adverse event may be related to the study intervention.

Serious adverse events, serious adverse reactions and unexpected serious adverse reactions are defined as any adverse event, adverse reaction or unexpected adverse reaction, respectively, that results in death, is life-threatening, requires hospitalisation or prolongation of existing hospitalisation, results in persistent or significant disability or incapacity, or results in a congenital anomaly or birth defect. All serious adverse events will be reported for this trial except hospitalisations due to a deterioration in mental state, as we anticipate a high proportion of our participants will be hospitalised for this reason during the trial. All hospitalisations or contact with home treatment teams will be recorded, for example on a service contact form.

In addition, the following assessments will be used to determine subject safety during the study at 3 and 6 months:

1. Corrected serum calcium levels (Ca^2+^) will be tested at baseline, at 3 and 6 months, and more frequently if nausea or vomiting occur.
2. At 3 and 6 months, a parathyroid hormone test will be performed on the blood sample if the corrected Ca ^2+^ level is above the normal range. If participants report nausea or vomiting between visits, they will be advised to contact their doctor for a Ca^2+^ blood test.
3. At baseline, female participants will undergo a urine dipstick pregnancy test (testing for beta human chorionic gonadotropin). Female participants of child-bearing age who refuse to take a pregnancy test at baseline will be excluded from participating in the trial. At each treatment visit (monthly for 6 months), the test will be performed again to determine pregnancy status. Pregnancy tests will not be undertaken by female participants who are permanently sterile or who are post-menopausal (no menses for 12 months without an alternative medical cause). If the urine test is positive, the participant will be withdrawn from the trial medication but encouraged to remain in the study. In the event that it is not possible to obtain a urine sample, a blood sample will be obtained and tested for beta human chorionic gonadotropin. No dose of IMP will be administered to female participants without first checking their pregnancy status (except for patients who are permanently sterile or post-menopausal, as described above).

Discontinuation of treatment will occur if a participant begins taking vitamin D doses above 400 IU per day, if they become pregnant, if their parathyroid hormone test is abnormal or at the discretion of the principal investigator.

### Dissemination

The research findings will be disseminated via peer-reviewed journals, conferences, internal reports and user group meetings and informed by the lived experience of our experts. When the project is complete, we will be able to provide all participants with a general summary of our research through a project newsletter.

## Discussion

There is a growing body of evidence that suggests vitamin D is neuroprotective [1, 2, 10] and that vitamin D deficiency is prevalent in those with schizophrenia [12, 16]. Longitudinally, the severity of symptoms in psychosis has also been associated with vitamin D concentration [24]. Lack of vitamin D is also associated with physical health problems, including adverse cardiovascular outcomes [50], and so may potentially compound the poor health status associated with psychotic disorders [51, 52]. Few studies have, however, examined whether vitamin D supplementation may improve outcomes among people with psychosis. Our study is the first rigorously designed trial to investigate the impact of vitamin D supplementation in early psychosis.

Our eligibility criteria include only patients who have the capacity to consent to the trial and thus, who have stable and minimal symptoms. We hypothesise that this will minimise the acute effect of the standard treatment on our outcome measures. We have elected not to restrict eligibility to people known to be vitamin D deficient, as it is not standard practice to test vitamin D levels on first presentation with psychosis. Introducing testing prior to study entry would alter clinical practice and introduce a novel clinical question on how to respond to the findings in the absence of applicable evidence. It would also limit the capacity of the study to determine whether there is benefit in supplementing all people with FEP with vitamin D. As we will be analysing baseline vitamin D levels after trial completion, we will be able to conduct a sub-analysis of this patient group.

A key strength of our study is its methodological rigour, since utilising a double-blind randomised controlled trial design removes potential biases inherent in cross-sectional research. The study is designed to take a practical approach and recruit from local clinics. This approach not only enhances the ecological validity of the outcomes but also augments existing connections between clinical and academic networks.

Vitamin D supplementation after FEP is as yet an unexplored area of research and clinical equipoise exists regarding its potential for benefit [53]. This trial will evaluate whether vitamin D may improve mental health indicators in patients with FEP, with a view to informing protocols for vitamin D in patients with FEP. Vitamin D supplementation is cheap, simple to access (e.g. over the counter), relatively safe and likely to be publicly acceptable [54]. Thus, even if vitamin D shows only small effects on clinical outcomes, vitamin D supplementation may offer a potentially effective treatment avenue for FEP patients.

### Trial status

The current protocol is version 10.1 (3 May 2019). The study protocol was written in accordance with the Standard Protocol Items: Recommendations for Interventional Trials (SPIRIT) guidelines. Table 1 lists the changes to the registered protocol (ISRCTN12424842). The study began recruiting in January 2016, and recruitment was completed on 14 June 2019. Follow-ups will continue until December 2019 and the results are expected to be available in 2020.

**Table 1:** Changes to the registered protocol

Protocol version	Description
2	Addition of new questionnaires including on level of sun exposure, frequency of eating food containing vitamin D, key service contacts, Fitzpatrick skin type, levels of physical activity (IPAQ) and duration of untreated psychosis.
3	Addition of second questionnaire assessing physical activity (SIMPAQ). Addition of PANSS assessment at month 3.
4	Changes to all study documents for clarification and to include site-specific information and logos.
5	The definition of the first episode of psychosis was changed from “6 months post-first presentation to services” to “3 years post-first presentation to services”.
6	Change in inclusion criteria to allow patients to take up to 400 IU per day of vitamin D in view of new Public Health England recommendations.
7	Change to inclusion criteria to allow patients aged up to 65 years to participate. Removal of anticonvulsants as an exclusion criterion. Remove hospitalisation due to a deterioration in mental health from the definition of a serious adverse event. Blood test for beta human chorionic gonadotropin was included if a urine test was not possible. Clarification for permitted dosing windows. Total study duration was reduced from 12 to 6 months.
8	Removal of exclusion criteria regarding anaemia, sickle cell anaemia and thalassemia.
9.1*	Clarification of outcome measures. Time between doses to be a minimum of 24 days. Clarification of window for collection of final assessment. Pregnancy test not required if participant is medically sterile or post-menopause. Vitamin D levels will be sent to GP at the end of the study. Phase II designation (not Phase IV).
9.2	Change in study end date to December 2019.
10.1*	Discontinuation of RNA sample collection. Increase in participant reimbursement. Clarifications to existing procedures regarding safety of the iPTH test, service contact form use, collection of adverse event details and attempts to contact participants for the follow-up.

*Protocol versions 9 and 10 were resubmitted as versions 9.1 and 10.1, respectively, after addressing comments from the MHRA and resubmitting to the research ethics committee.

*IPAQ* International Physical Activity Questionnaire, *PANSS* Positive and Negative Symptom Scale, *SIMPAQ* Simple Physical Activity Questionnaire

## Supplementary information

**Additional file 1.** SPIRIT checklist for the protocol of a clinical trial.

## Data Availability

Not applicable.

## Abbreviations

25OHD: 25-hydroxyvitamin D
CDS: Calgary Depression Scale
CRP: C-reactive protein
FEP: First episode psychosis
GAF: Global Assessment of Functioning
HbA1c: Haemoglobin A1c
HRA: Health Research Authority
ICD-10: International Classification of Diseases, 10th Edition
ICH-GCP: International Council on Harmonisation’s Good Clinical Practice Guideline
IMP: Investigational medicinal product
IPAQ: International Physical Activity Questionnaire
IU: International units
MHRA: Medicines and Healthcare Products Regulatory Agency
NHS: National Health Service
NIHR: National Institute for Health Research
NOS: Nottingham Onset Schedule
OPCRIT: Operational Criteria
PANSS: Positive and Negative Syndrome Scale
SIMPAQ: Simple Physical Activity Questionnaire
TSC: Trial steering committee

## References

[1] DeLuca GC, Kimball SM, Kolasinski J, Ramagopalan SV, Ebers GC (2013). Review: the role of vitamin D in nervous system health and disease. Neuropathol Appl Neurobiol.

[2] Wrzosek M, Lukaszkiewicz J, Wrzosek M, Jakubczyk A, Matsumoto H, Piatkiewicz P (2013). Vitamin D and the central nervous system. Pharmacol Rep.

[3] McCann JC, Ames BN (2008). Is there convincing biological or behavioral evidence linking vitamin D deficiency to brain dysfunction?. FASEB J.

[4] Eyles DW, Burne TH, McGrath JJ (2013). Vitamin D, effects on brain development, adult brain function and the links between low levels of vitamin D and neuropsychiatric disease. Front Neuroendocrinol.

[5] Kesby JP, Turner KM, Alexander S, Eyles DW, McGrath JJ, Burne THJ (2017). Developmental vitamin D deficiency alters multiple neurotransmitter systems in the neonatal rat brain. Int J Dev Neurosci.

[6] Groves NJ, Kesby JP, Eyles DW, McGrath JJ, Mackay-Sim A, Burne TH (2013). Adult vitamin D deficiency leads to behavioural and brain neurochemical alterations in C57BL/6J and BALB/c mice. Behav Brain Res.

[7] Lima LAR, Lopes MJP, Costa RO, Lima FAV, Neves KRT, Calou IBF (2018). Vitamin D protects dopaminergic neurons against neuroinflammation and oxidative stress in hemiparkinsonian rats. J Neuroinflammation.

[8] Feron F, Burne TH, Brown J, Smith E, McGrath JJ, Mackay-Sim A (2005). Developmental Vitamin D_3_ deficiency alters the adult rat brain. Brain Res Bull.

[9] Balden R, Selvamani A, Sohrabji F (2012). Vitamin D deficiency exacerbates experimental stroke injury and dysregulates ischemia-induced inflammation in adult rats. Endocrinology.

[10] Vuillermot S, Luan W, Meyer U, Eyles D (2017). Vitamin D treatment during pregnancy prevents autism-related phenotypes in a mouse model of maternal immune activation. Mol Autism.

[11] Suzuki M, Yoshioka M, Hashimoto M, Murakami M, Noya M, Takahashi D (2013). Randomized, double-blind, placebo-controlled trial of vitamin D supplementation in Parkinson disease. Am J Clin Nutr.

[12] Cui X, Groves NJ, Burne TH, Eyles DW, McGrath JJ (2013). Low vitamin D concentration exacerbates adult brain dysfunction. Am J Clin Nutr.

[13] Wang C, Zhang Y (2017). Season of birth and schizophrenia: Evidence from China. Psychiatry Res.

[14] Davies G, Welham J, Chant D, Torrey EF, McGrath J (2003). A systematic review and meta-analysis of Northern Hemisphere season of birth studies in schizophrenia. Schizophr Bull.

[15] Cantor-Graae E, Selten JP (2005). Schizophrenia and migration: a meta-analysis and review. Am J Psychiatry.

[16] McGrath JJ, Burne TH, Feron F, Mackay-Sim A, Eyles DW (2010). Developmental vitamin D deficiency and risk of schizophrenia: a 10-year update. Schizophr Bull.

[17] Eyles DW, Trzaskowski M, Vinkhuyzen AAE, Mattheisen M, Meier S, Gooch H (2018). The association between neonatal vitamin D status and risk of schizophrenia. Sci Rep.

[18] Tolppanen AM, Sayers A, Fraser WD, Lewis G, Zammit S, McGrath J (2012). Serum 25-hydroxyvitamin D_3_ and D_2_ and non-clinical psychotic experiences in childhood. PLoS One.

[19] Valipour G, Saneei P, Esmaillzadeh A (2014). Serum vitamin D levels in relation to schizophrenia: a systematic review and meta-analysis of observational studies. J Clin Endocrinol Metab.

[20] Hedelin M, Lof M, Olsson M, Lewander T, Nilsson B, Hultman CM (2010). Dietary intake of fish, omega-3, omega-6 polyunsaturated fatty acids and vitamin D and the prevalence of psychotic-like symptoms in a cohort of 33,000 women from the general population. BMC Psychiatry.

[21] The National Institute for Health and Care Excellence. Clinical Knowlege Summaries. Vitamin D deficiency in adults - treatment and prevention. London: The National Institute for Health and Care Excellence; 2018. Available from: https://cks.nice.org.uk/vitamin-d-deficiency-in-adults-treatment-and-prevention#!topicSummary.

[22] Lally J, Gardner-Sood P, Firdosi M, Iyegbe C, Stubbs B, Greenwood K (2016). Clinical correlates of vitamin D deficiency in established psychosis. BMC Psychiatry.

[23] Crews M, Lally J, Gardner-Sood P, Howes O, Bonaccorso S, Smith S (2013). Vitamin D deficiency in first episode psychosis: a case-control study. Schizophr Res.

[24] Lally John, Ajnakina Olesya, Singh Nidhita, Gardner-Sood Poonam, Stubbs Brendon, Stringer Dominic, Di Forti Marta, David Anthony S., Smith Shubulade, Murray Robin M., Howes Oliver D., Gaughran Fiona (2019). Vitamin D and clinical symptoms in First Episode Psychosis (FEP): A prospective cohort study. Schizophrenia Research.

[25] Firth J, Carney R, Stubbs B, Teasdale SB, Vancampfort D, Ward PB (2018). Nutritional Deficiencies and Clinical Correlates in First-Episode Psychosis: A Systematic Review and Meta-analysis. Schizophr Bull.

[26] Thornicroft G (2011). Physical health disparities and mental illness: the scandal of premature mortality. Br J Psychiatry.

[27] Gardner-Sood P, Lally J, Smith S, Atakan Z, Ismail K, Greenwood KE (2015). Cardiovascular risk factors and metabolic syndrome in people with established psychotic illnesses: baseline data from the IMPaCT randomized controlled trial. Psychol Med.

[28] Vancampfort D, Correll CU, Galling B, Probst M, De Hert M, Ward PB (2016). Diabetes mellitus in people with schizophrenia, bipolar disorder and major depressive disorder: a systematic review and large scale meta-analysis. World Psychiatry.

[29] Correll CU, Solmi M, Veronese N, Bortolato B, Rosson S, Santonastaso P (2017). Prevalence, incidence and mortality from cardiovascular disease in patients with pooled and specific severe mental illness: a large-scale meta-analysis of 3,211,768 patients and 113,383,368 controls. World Psychiatry.

[30] Hypponen E, Boucher BJ, Berry DJ, Power C (2008). 25-hydroxyvitamin D, IGF-1, and metabolic syndrome at 45 years of age: a cross-sectional study in the 1958 British Birth Cohort. Diabetes.

[31] Lucato P, Solmi M, Maggi S, Bertocco A, Bano G, Trevisan C (2017). Low vitamin D levels increase the risk of type 2 diabetes in older adults: A systematic review and meta-analysis. Maturitas.

[32] Bassuk SS, Manson JE, Lee IM, Cook NR, Christen WG, Bubes VY (2016). Baseline characteristics of participants in the VITamin D and OmegA-3 TriaL (VITAL). Contemp Clin Trials.

[33] Tiangga E, Gowda A, Dent JA (2008). Vitamin D deficiency in psychiatric in-patients and treatment with daily supplements of calcium and ergocalciferol. Psychiatr Bull.

[34] Dealberto MJ (2013). Clinical symptoms of psychotic episodes and 25-hydroxy vitamin D serum levels in black first-generation immigrants. Acta Psychiatr Scand.

[35] Thakurathi N, Stock S, Oppenheim CE, Borba CP, Vincenzi B, Seidman LJ (2013). Open-label pilot study on vitamin D (3) supplementation for antipsychotic-associated metabolic anomalies. Int Clin Psychopharmacol.

[36] Krivoy A, Onn R, Vilner Y, Hochman E, Weizman S, Paz A (2017). Vitamin D Supplementation in Chronic Schizophrenia Patients Treated with Clozapine: A Randomized, Double-Blind, Placebo-controlled Clinical Trial. EBioMedicine.

[37] ICH Expert Working Group (1996). Guideline for Good Clinical Practice.

[38] Kay SR, Fiszbein A, Opler LA (1987). The positive and negative syndrome scale (PANSS) for schizophrenia. Schizophr Bull.

[39] Hall RC (1995). Global assessment of functioning. A modified scale. Psychosomatics.

[40] Jones SH, Thornicroft G, Coffey M, Dunn G (1995). A brief mental health outcome scale-reliability and validity of the Global Assessment of Functioning (GAF). Br J Psychiatry.

[41] Vatnaland T, Vatnaland J, Friis S, Opjordsmoen S (2007). Are GAF scores reliable in routine clinical use?. Acta Psychiatr Scand.

[42] Hilsenroth MJ, Ackerman SJ, Blagys MD, Baumann BD, Baity MR, Smith SR (2000). Reliability and validity of DSM-IV axis V. Am J Psychiatry.

[43] Startup M, Jackson MC, Bendix S (2002). The concurrent validity of the Global Assessment of Functioning (GAF). Br J Clin Psychol.

[44] Addington D, Addington J, Schissel B (1990). A depression rating scale for schizophrenics. Schizophr Res.

[45] Addington D, Addington J, Maticka-Tyndale E, Joyce J (1992). Reliability and validity of a depression rating scale for schizophrenics. Schizophr Res.

[46] Gaughran Fiona, Stahl Daniel, Stringer Dominic, Hopkins David, Atakan Zerrin, Greenwood Kathryn, Patel Anita, Smith Shubulade, Gardner-Sood Poonam, Lally John, Heslin Margaret, Stubbs Brendon, Bonaccorso Stefania, Kolliakou Anna, Howes Oliver, Taylor David, Forti Marta Di, David Anthony S., Murray Robin M., Ismail Khalida (2019). Effect of lifestyle, medication and ethnicity on cardiometabolic risk in the year following the first episode of psychosis: prospective cohort study. British Journal of Psychiatry.

[47] Patel JV, Chackathayil J, Hughes EA, Webster C, Lip GY, Gill PS (2013). Vitamin D deficiency amongst minority ethnic groups in the UK: a cross sectional study. Int J Cardiol.

[48] Information Commissioner’s Office (2018). Guide to the General Data Protection Regulation (GDPR) UK.

[49] White IR, Royston P, Wood AM (2011). Multiple imputation using chained equations: Issues and guidance for practice. Stat Med.

[50] Melamed ML, Michos ED, Post W, Astor B (2008). 25-hydroxyvitamin D levels and the risk of mortality in the general population. Arch Intern Med.

[51] Berk M, Jacka FN, Williams LJ, Ng F, Dodd S, Pasco JA (2008). Is this D Vitamin to Worry About? Vitamin D Insufficiency in an Inpatient Sample. Aust N Z J Psychiatry.

[52] Leucht S, Burkard T, Henderson J, Maj M, Sartorius N (2007). Physical illness and schizophrenia: a review of the literature. Acta Psychiatr Scand.

[53] Firth J, Teasdale SB, Allott K, Siskind D, Marx W, Cotter J (2019). The efficacy and safety of nutrient supplements in the treatment of mental disorders: a meta-review of meta-analyses of randomized controlled trials. World Psychiatry.

[54] Jessiman T, Cameron A, Wiggins M, Lucas PJ (2013). A qualitative study of uptake of free vitamins in England. Arch Dis Child.

